# The trans-Golgi SNARE syntaxin 10 is required for optimal development of *Chlamydia trachomatis*

**DOI:** 10.3389/fcimb.2015.00068

**Published:** 2015-09-25

**Authors:** Andrea L. Lucas, Scot P. Ouellette, Emily J. Kabeiseman, Kyle H. Cichos, Elizabeth A. Rucks

**Affiliations:** Division of Basic Biomedical Sciences, Sanford School of Medicine, University of South DakotaVermillion, SD, USA

**Keywords:** *Chlamydia trachomatis*, syntaxin 10, chlamydial development, lipid trafficking, *trans*-Golgi SNARE

## Abstract

*Chlamydia trachomatis*, an obligate intracellular pathogen, grows inside of a vacuole, termed the inclusion. Within the inclusion, the organisms differentiate from the infectious elementary body (EB) into the reticulate body (RB). The RB communicates with the host cell through the inclusion membrane to obtain the nutrients necessary to divide, thus expanding the chlamydial population. At late time points within the developmental cycle, the RBs respond to unknown molecular signals to redifferentiate into infectious EBs to perpetuate the infection cycle. One strategy for *Chlamydia* to obtain necessary nutrients and metabolites from the host is to intercept host vesicular trafficking pathways. In this study we demonstrate that a *trans*-Golgi soluble *N*-ethylmaleimide–sensitive factor attachment protein (SNARE), syntaxin 10, and/or syntaxin 10-associated Golgi elements colocalize with the chlamydial inclusion. We hypothesized that *Chlamydia* utilizes the molecular machinery of syntaxin 10 at the inclusion membrane to intercept specific vesicular trafficking pathways in order to create and maintain an optimal intra-inclusion environment. To test this hypothesis, we used siRNA knockdown of syntaxin 10 to examine the impact of the loss of syntaxin 10 on chlamydial growth and development. Our results demonstrate that loss of syntaxin 10 leads to defects in normal chlamydial maturation including: variable inclusion size with fewer chlamydial organisms per inclusion, fewer infectious progeny, and delayed or halted RB-EB differentiation. These defects in chlamydial development correlate with an overabundance of NBD-lipid retained by inclusions cultured in syntaxin 10 knockdown cells. Overall, loss of syntaxin 10 at the inclusion membrane negatively affects *Chlamydia*. Understanding host machinery involved in maintaining an optimal inclusion environment to support chlamydial growth and development is critical toward understanding the molecular signals involved in successful progression through the chlamydial developmental cycle.

## Introduction

*Chlamydia* are obligate intracellular pathogens and multiply within mucosal epithelial cells. The organisms grow inside host cells within an enclosed membrane bound vacuole termed an inclusion. *C. trachomatis* infections negatively impact human health worldwide and are associated with both genital (serovars D–K and LGV L1-3) and ocular infections (serovars A–C); (Schachter, 1999; Datta et al., 2007; Centers for Disease Control and Prevention, 2012). Notably, *C. trachomatis* is the most commonly reported bacterial sexually transmitted disease in the United States. Chlamydial sexually transmitted infections are predominantly asymptomatic, which perpetuates the spread of disease to unsuspecting partners. Prolonged chlamydial infection, often associated with asymptomatic disease, can result in infertility problems for women and an increased risk of acquiring HPV or HIV (Centers for Disease Control and Prevention, 2012).

All *Chlamydia* spp. have a biphasic developmental cycle in which the organisms alternate between two different developmental forms (Abdelrahman and Belland, 2005). The infectious elementary body (EB) rapidly differentiates into a replicative, non-infectious reticulate body (RB). The developmental cycle is completed when RBs redifferentiate into infectious EBs and exit the host cell (Ward, 1988; Abdelrahman and Belland, 2005). Initially the inclusion membrane is composed of primarily host cell plasma membrane but becomes studded with chlamydial proteins secreted by a type III secretion system (Fields et al., 2003). This allows the inclusion to disassociate from the endocytic pathway to avoid fusion with the lysosome (Heinzen et al., 1996; Taraska et al., 1996; Van Ooij et al., 1997; Fields et al., 2003; Scidmore et al., 2003). The entirety of the development cycle occurs within the confines of a pathogen-specified parasitic organelle termed the chlamydial inclusion (Moore and Ouellette, 2014).

*Chlamydia* species have evolved closely with their host resulting in significant genome reduction. *Chlamydia* synthesize some of their own nutrients, amino acids, and nucleic acids, but, where chlamydial biosynthetic pathways have been lost, the organisms acquire essential metabolites from the host (Wylie et al., 1997; Stephens et al., 1998; McClarty, 2004; Elwell and Engel, 2012). For example, RBs lack the ability to actively synthesize some of the lipids which are found in their cell walls such as glycerophospholipids and phosphatidylcholine, suggesting they acquire these lipids from the host (Wylie et al., 1997). Despite the great metabolic needs of chlamydial organisms, the host cell is not overtly stressed by a chlamydial infection, suggesting that *Chlamydia* work in collaboration with the host cell in order to obtain the necessary and required nutrients which support chlamydial growth and development (Moore and Ouellette, 2014). To this end, chlamydial organisms have developed the ability to redirect necessary metabolites to the inclusion (Hackstadt et al., 1995; Heinzen et al., 1996). Golgi-derived lipid trafficking to the inclusion is, in part, vesicular in nature (Hackstadt et al., 1996); however, non-vesicular mechanisms have also been demonstrated (Cocchiaro et al., 2008; Derré et al., 2011; Elwell et al., 2011). A polarized cell model of chlamydial infection determined that *Chlamydia* preferentially intercept Golgi-derived vesicles in route to the basolateral plasma membrane (Moore et al., 2008). In order to define eukaryotic and chlamydial inclusion membrane fusion events, we examined host proteins that govern membrane fusion along basolateral trafficking pathways. SNARE proteins syntaxin 6 and VAMP4, but not syntaxins 4, 5, or 16, localize to the chlamydial inclusion (Moore et al., 2011; Kabeiseman et al., 2013). These observations emphasize the specific interaction that the inclusion membrane has with host SNARE proteins.

We hypothesize that *Chlamydia* utilizes components of the eukaryotic molecular machinery to facilitate membrane fusion events at the chlamydial inclusion to optimize nutrient acquisition. In testing this hypothesis, we demonstrate that syntaxin 10 localizes to the chlamydial inclusion. Further, loss of syntaxin 10 due to siRNA knockdown results in detrimental delays to chlamydial growth and development. These data suggest that syntaxin 10 contributes to a cellular process required to support an optimum growing environment for *Chlamydia*.

## Materials and methods

### Cell culture and chlamydial organisms

HeLa 229 cells (ATCC-CCL-2.1; Manassas, VA) were cultured as previously described (Kabeiseman et al., 2013). HEp2 cells (Harlan Caldwell, Rocky Mountain Laboratories, Hamilton, MT) were maintained in Iscove's Modified Dulbecco's medium (Gibco/Life Technologies, Carlsbad, CA) supplemented with 10% fetal bovine serum (GE Healthcare Hyclone, Logan, UT). *Chlamydia trachomatis* L2 were propagated in HeLa cells or HEp2 cells and infectious elementary bodies (EBs) were isolated, enumerated, and stocks were stored at -80°C as previously described (Furness et al., 1960; Caldwell et al., 1981; Scidmore, 2005).

### 3XFLAG-syntaxin 10 transfections and bacterial infections

Syntaxin 10 (GenBank accession number CR457110) cDNA (Eurofins MWG Operon; Huntsville, Alabama) served as a template to clone syntaxin 10. The forward primer (5′- ggggggaattcaatgtctctcgaagacccctttt-3′) contained a 5′ EcoRI site (all caps, underlined), and the reverse primer (5′- gggggGGATCCtcagagagagaatagtaagatgagaa-3′) contained a 3′ BamHI site (all caps, underlined), which were used to subclone *syntaxin 10* into the multiple cloning site of the p3XFLAG-CMV 7.1 expression vector (Sigma; St. Louis, MO). Sequence was verified by Eurofins MWG Operon, Huntsville, AL. 100 ng of plasmid DNA was transfected with Lipofectamine 2000 Transfection (Life Technologies, Carlsbad, CA) and Opti-MEM (Life Technologies), according to the manufacturer's protocol. Cells were then infected with *Chlamydia* as described previously (Scidmore, 2005).

### Immunofluorescence microscopy

HeLa cells were seeded onto 12 mm glass coverslips 24 h before transfection of the appropriate construct, followed by infection with *C. trachomatis* serovar L2. 16–18 h post-infection, cells were fixed in 4% paraformaldehyde (Acros Organics/Thermo Scientific; Logan, UT) and permeabilized with 0.5% TritonX-100 for 5 min. Coverslips were incubated with primary antibody: mouse anti-FLAG M2 (Sigma Aldrich), rabbit anti-IncA (Ted Hackstadt, Rocky Mountain Laboratories, Hamilton, MT), rabbit anti-IncG (Ted Hackstadt), or rabbit anti-giantin (Covance; Emeryville, CA), followed by incubation with the appropriate secondary antibody conjugated to DyLight fluors (Jackson ImmunoResearch Laboratories; West Grove, PA). Coverslips were mounted on glass microscope slides using Prolong Gold mounting reagent (Life Technologies). Slides were visualized with an 60X objective and 2x zoom using an Olympus Fluoview 1000 Laser Scanning Confocal Microscope.

### siRNA transfection

Silencer select siRNA (Life Technologies) against syntaxin 10 (Stx10); (s16535) and non-targeting control (NT); (4390843) were used at a final concentration of 10 nM. All siRNA clones were validated by Life Technologies according to their procedures. Three different siRNA clones against Stx10 were used (ID number-137195, ID number-137196, and ID number-137197) to quantify chlamydial infectious progeny at 44 h of infection. Based on similar results between all Stx10 siRNAs (data not shown), Stx10 ID number-137197 was used for all subsequent siRNA experiments. siRNA was delivered to HeLa cells via reverse transfection using Lipofectamine RNAiMAX siRNA Transfection Reagent and Opti-MEM media (Life Technologies), following the manufacturer's protocol. For all siRNA knockdown experiments, monolayers were infected with *C. trachomatis* serovar L2 48 h after siRNA transfection. Efficiency of knockdown was confirmed by Western blot and densitometry analysis using Odyssey Infrared Imaging System using Image Studio imaging software (LI-COR, Lincoln, NE). Only samples achieving 70% or greater knockdown efficiency were used in subsequent studies.

### Quantification of inclusion formation and chlamydial infectious progeny

Through the course of our studies, we recognized that syntaxin 10 knockdown cells do not divide at the same rate as cells transfected with non-targeting siRNA. Therefore, on the day of infection, cells were enumerated in order to infect all monolayers at a multiplicity of infection (moi) of 0.5 bacteria per eukaryotic cell. To assess inclusion formation, monolayers were fixed and processed for immunofluorescence to detect chlamydial inclusions and host nuclei. 7–10 fields of view from 3 separate coverslips were enumerated. Infectious progeny produced at 24, 44, and 67 h post-infected were harvested and replated onto HEp2 cells, essentially as previously described (Kabeiseman et al., 2013). Serial dilutions of infectious progeny were plated in triplicate and 10 fields of view from each coverslip were enumerated. Because there were greater numbers of cells in the wells transfected with non-targeting siRNA, a greater number of bacteria were used to reach an moi of 0.5. Therefore, to correct for this disparity, the inclusion forming units (IFU) obtained from wells transfected with non-targeting siRNA were divided by the increased number of bacteria compared to the inoculum of syntaxin 10 siRNA-treated cells. Data shown are from one experiment, but are consistent with all 3 independent experiments. These values were calculated and graphed using GraphPad Prism 6 Software (GraphPad Software, La Jolla, CA), as described below. As the monolayer in the NT siRNA treated cells was destroyed at 67 h post-infection, the knockdown of syntaxin 10 at the 67 h time point was determined by comparing the ratio of syntaxin 10 to GAPDH to the 44 h time point of the NT siRNA treated cells.

### Chlamydial protein expression by SDS-PAGE and western blot analysis

siRNA transfected cells were infected with *Chlamydia* as described above. Samples were separated on an SDS-12%PAGE and transferred using an iBlot transfer apparatus (Life Technologies). Primary antibodies used in this study include: rabbit anti-syntaxin 10 (Abgent; SanDiego, CA), mouse anti-GAPDH (EMD Millipore; Darmstadt, Germany), rabbit anti-Hc1 (Ted Hackstadt), rabbit anti-OmcB (Thomas Hatch, University of Tennessee Health Science Center, Memphis, TN), and mouse anti-HSP60 (Rick Morrison, Department of Microbiology and Immunology, University of Arkansas for Medical Sciences, Little Rock, AR). The primary antibodies were detected using appropriate anti-mouse or anti-rabbit IgG secondary antibodies conjugated with IRDye 700 or IRDye 800 dyes (LI-COR). The blots were scanned and analyzed by densitometry with an Odyssey Infrared Imaging System using Image Studio imaging software (LI-COR). To quantitate OmcB and Hc1 protein levels, densitometry values were normalized to cHSP60, which was first normalized to host GAPDH. Data are representative of two independent experiments and results are expressed as mean and standard error of the mean, calculated by GraphPad Prism 6 software.

### Transmission electron microscopy (TEM)

Syntaxin 10 siRNA or non-targeting control siRNA transfected monolayers were infected with *C. trachomatis* serovar L2 for 36 h. Infected monolayers were collected and fixed in 2% EM-grade paraformaldehyde plus 2.5% EM-grade glutaraldehyde (Polysciences Inc., Warrington, PA) in 100 mM sodium phosphate buffer (Sigma Aldrich). Cells were processed for TEM as described previously (Beatty, 2006). TEM images were taken for two independent experiments. TEM images were used to quantify total numbers of organisms per inclusion, percentage of developmental forms in each inclusion and inclusion diameter. The diameter measurement was taken at the widest part of each inclusion. The results are displayed as μm units, based on the scale set from the electron microscope. All means and standard error of the means were calculated using GraphPad Prism 6 software.

### Live cell imaging

At the indicated time points after infection with *C. trachomatis* serovar L2, cells were labeled with 5 μM 6-((*N*-(7-nitrobenz-2-oxa-1, 3-diazol-4-yl)amino)hexanoyl)sphingosine (C_6_-NBD-ceramide); (Life Technologies) as described previously (Hackstadt et al., 1995; Moore, 2012; Kabeiseman et al., 2013). Phase contrast and fluorescent live cell images were acquired at the indicated time points post back-exchange using the 40X phase objective with the Axiovert 200 M Imager with the AxioCam HRm camera (Carl Zeiss Microscopy, LLC; Thornwood, NY). To quantify the relative brightness of the inclusions (per area of the individual inclusions) cultured in non-targeting control (NT) or syntaxin 10 (stx10) siRNA-treated cells, cells were infected with *C. trachomatis* for 30 h, labeled with C_6_-NBD-ceramide and back-exchanged for 1.5 h. Coverslips were mounted onto glass slides and imaged at 40 ms (NT) or 20 ms (Stx10) exposure times (to prevent saturation) with an Olympus BX 60 fluorescent scope (60X magnification) and images taken with a Nikon DS-Qi1Mc camera. 20 images were taken from two independent experiments, each performed in duplicate (40 images were processed for each independent experiment). The fluorescent intensity (integrated density) and area of the inclusion were determined with ImageJ v1.48 (National Institutes of Health, Bethesda, MD). Measuring the integrated density and area of the “brightest” non-inclusion-associated background and subtracting that amount from inclusion measurements corrected all measurements for background fluorescence. Mean and standard error of the mean were calculated and graphed using GraphPad Prism 6 software.

### Statistics and image production

All quantification and statistical analysis of data were performed with GraphPad Prism 6 Software. Statistical analyses used include an ordinary one-way analysis of variance (ANOVA) with a Tukey's multiple comparison *post-hoc* test, or Student's *t*-test, as appropriate. The use of specific statistical tests is indicated in the associated figure legends. All figures were constructed using Adobe Photoshop CS5 (Adobe Systems Incorporated, San Jose, CA). Modifications to images include adjustment to color balance in fluorescent images, applied equally to all images in a single figure, with the exception of live cell images appearing in **Figure 5A** to which no adjustments were made. Brightness and contrast were adjusted in Western blot images.

## Results

### Colocalization of syntaxin 10 with the chlamydial inclusion

**Figure 1:**
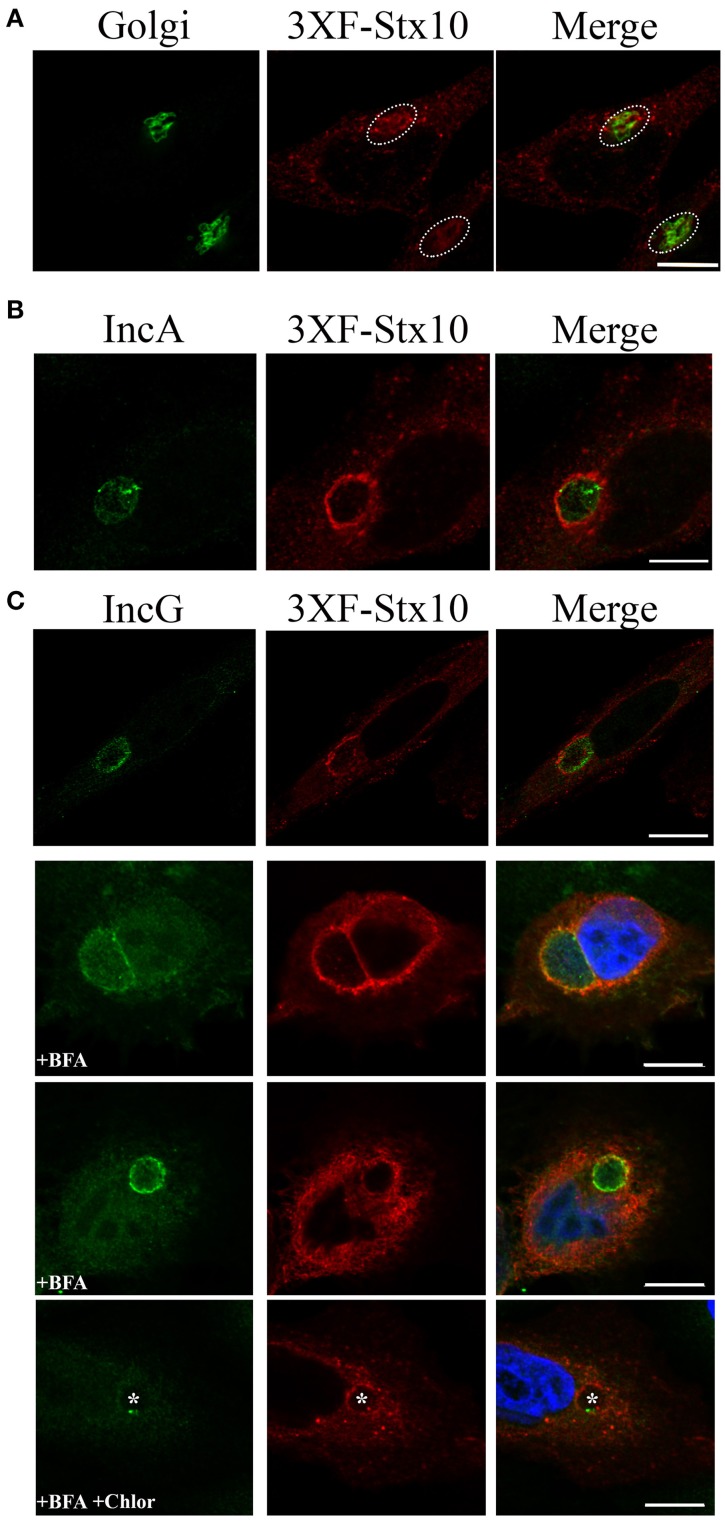
**Syntaxin 10 localization to the chlamydial inclusion. (A)** HeLa cells were transfected with 3XFLAG-syntaxin 10 (3XF-stx10) for 24 h prior to fixation and processing for imaging. The Golgi (outlined in white) was detected with a rabbit anti-giantin antibody; 3XF-Stx10 was detected using a mouse anti-FLAG M2 antibody. **(B,C)** HeLa or HEp2 cells transfected with 3XF-stx10 were infected for 16–18 h with *C. trachomatis* serovar L2, prior to fixation and processing for imaging. The inclusion membrane was detected using either a rabbit anti- IncA **(B)** (Additional images provided in Supplemental Figure 1) or IncG **(C)** antibody; 3XF-Stx10 was detected as above. To distinguish 3XF-Stx10 from surrounding cell structures, some samples were treated with brefeldin A (BFA) to collapse the surrounding Golgi. To examine if chlamydial protein synthesis was required for 3XF-Stx10 localization, infected monolayers were treated with 200 μg/ml chloramphenicol (Chlor) for an additional 24 h prior to fixation. In chloramphenicol treated cells, white asterisks indicate inclusions. All images were acquired using an Olympus Fluoview 1000 Laser Scanning Confocal Microscope with a 60X objective and 2x zoon. These results are representative of at least 3 independent experiments. White bars = 10 μm.

Previous studies demonstrated that trans-Golgi SNARE proteins syntaxin 6 and VAMP4 localize to the chlamydial inclusion (Moore et al., 2011; Kabeiseman et al., 2013). We hypothesize that *Chlamydia* recruit specific SNARE proteins to help the chlamydial inclusion maintain an optimal growing environment for the pathogens. Missing from these previous analyses was an understanding of whether syntaxin 10, another trans-Golgi SNARE, localized to the chlamydial inclusion. We initially tried to visualize endogenous syntaxin 10 by indirect immunofluorescence, but commercially available antibodies were not suitable for this application. Therefore, for these studies, we transfected HeLa cells with a 3XFLAG-syntaxin 10 construct, which localized in vesicular-like structures throughout the cell and within the Golgi apparatus (Figure 1A). By confocal microscopy, exogenously expressed 3XFLAG-syntaxin 10 colocalized with two inclusion membrane markers: IncA and IncG (Figures 1B,C). What is apparent in these images is the vesicular nature of 3XFLAG-syntaxin 10 structures at the inclusion. 3XFLAG-syntaxin 10 does not form a distinct rim, as other eukaryotic proteins that localize to the chlamydial inclusion. Rather, it resembles a collection of vesicles, presumably Golgi-elements, since syntaxin 10 is strongly associated with the trans-Golgi network. Due to the localization pattern of syntaxin 10, the timing of the localization of 3XFLAG-syntaxin 10 at early time points post-infection is difficult to determine, but it likely occurs at some point between 8 and 14 h post-infection and remains associated with the inclusion beyond 36 h post-infection (Supplemental Figure 1). To distinguish 3XFLAG-syntaxin 10 that localized to the inclusion from surrounding cellular structures, cells were treated with 1 μg/ml of brefeldin A (BFA), which collapses the Golgi into the ER (Lippincott-Schwartz et al., 1989), for 2 h prior to fixation. BFA treatment did not eliminate the localization of 3XFLAG-syntaxin 10 with the inclusion, indicating that the localization of 3XFLAG-syntaxin 10 is not happenstance due to the inclusion's proximity with the Golgi (Figure 1C). As indicated in Figure 1C, association of these syntaxin 10 positive structures with the inclusion likely stabilizes the structures from the effects of BFA. Notably, inhibition of chlamydial protein synthesis at 18 h post-infection by chloramphenicol did not abolish the localization of 3XFLAG-syntaxin 10 to the chlamydial inclusion (Figure 1C, last row). These data indicate that once syntaxin 10 or syntaxin 10-positive structures are trafficked to the inclusion that the interaction is likely with a stable (i.e., low turnover) chlamydial protein. We were unable to determine if treatment of infected monolayers with chloramphenicol during early time points of infection inhibits localization of syntaxin 10 or syntaxin 10 positive structures with the inclusion (Supplemental Figure 1A).

### The effect of syntaxin 10 knockdown on inclusion and chlamydial growth and development

**Figure 2:**
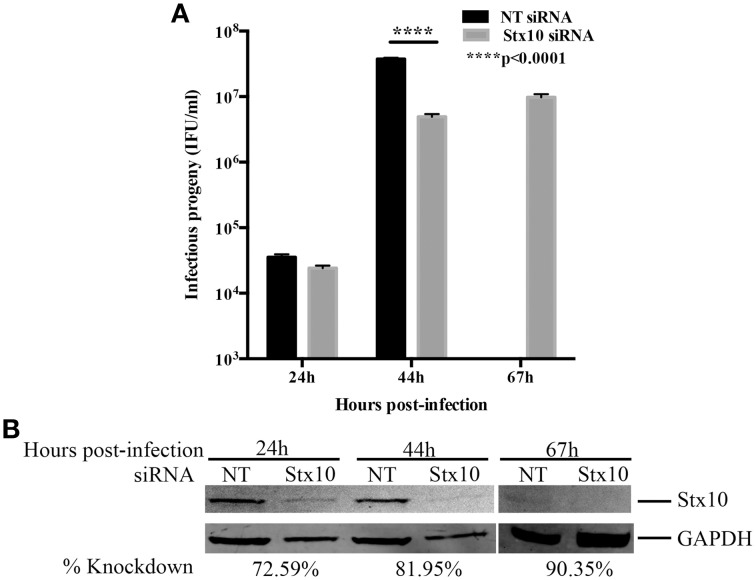
**Effect of siRNA knockdown of syntaxin 10 on inclusion development and chlamydial infectious progeny. (A)** Syntaxin 10 (Stx10) or NT (non-targeting, control) siRNA-treated HeLa cells were infected at an MOI of 0.5 with *C. trachomatis* serovar L2 for 24, 44, or 67 h. Monolayers were lysed in dH_2_O, then serial dilutions were replated onto a fresh monolayer of cells. These cells were fixed and processed to enumerate inclusions. Inclusions were counted using a 20X objective and values are expressed as mean and standard error of the mean and then analyzed with an ordinary One-Way ANOVA with a Tukey's multiple comparison test in GraphPad Prism 6 software. Data are representative of 3 independent experiments. **(B)** Corresponding Western blot of cell lysates harvested at the time points described above to test for efficient knockdown of syntaxin 10 (stx10) compared to loading control GAPDH.

The pattern of localization of syntaxin 10 to the chlamydial inclusion membrane suggested that this protein has a function for *Chlamydia*. To understand the relationship of syntaxin 10 with the Golgi, we initially used siRNA to knockdown syntaxin 10 and examined Golgi morphology (using Golgi protein, Giantin) around the chlamydial inclusion (Supplemental Figure 2). In control cells (cells transfected with non-targeting siRNA), the Golgi is condensed and encircles the chlamydial inclusion, as previously characterized (Heuer et al., 2009). In syntaxin 10 knockdown cells, the Golgi retains a discernable vesicular structure, but the tight association with the inclusion is partially lost. This suggests a potential role for syntaxin 10 and/or associated interacting protiens (i.e., Incs) in anchoring the Golgi to the inclusion. In the context of our working hypothesis, these data support a role for syntaxin 10 in contributing to an optimal environment for chlamydial development. We cannot readily distinguish between vesicular trafficking defects or “relaxed” Golgi effects on chlamydial development. However, previous studies have demonstrated that loss of Golgi morphology does not negatively impact chlamydial development (Hackstadt et al., 1996). As a first step in understanding the function of syntaxin 10 in chlamydial growth and development, we began by assessing the ability of organisms to produce infectious progeny 24, 44, and 67 h post-infection (Figure 2A). In a typical serovar L2 developmental cycle (organisms grown in HeLa or HEp2 cells), rapid division of RBs occurs between 8 and 16 h post-infection, with RB to EB differentiation occurring 24 to 36 h post-infection. Maximal RB to EB transition occurs between 42 and 48 h post-infection, with subsequent monolayer destruction due to maximal EB release occurring at or after 50+ h of infection (Ward, 1988; Dessus-Babus et al., 2008). Consistent with the progression of the normal chlamydial developmental cycle, there are low numbers of infectious progeny produced in both siRNA treatment groups (NT: 3.54 × 10^4^ ± 3.97 × 10^3^; Stx10: 2.41 × 10^4^ ± 2.24 × 10^3^) after 24 h of infection (mid-developmental cycle), indicating that chlamydial development is not altered due to depletion of syntaxin 10 at this time point post-infection. However, after 44 h of infection (late developmental cycle), there is a statistically and biologically significant ~10-fold decrease in infectious progeny obtained from *Chlamydia* grown in syntaxin 10 siRNA-treated cells (4.93 × 10^6^ ± 4.85 × 10^5^) vs. control cells (3.75 × 10^7^ ± 1.62 × 10^6^). Also apparent in these data is the 1000-fold increase of progeny produced in control cells between the 24 and 44 h time points vs. the 200-fold increase in progeny produced in syntaxin 10 knockdown cells within the same time frame. Therefore, organisms cultivated in the absence of syntaxin 10 demonstrated a 5-fold lower production rate of infectious progeny. We also assessed if a longer incubation of *Chlamydia* in syntaxin 10 siRNA-treated cells would yield more progeny. At 67 h post-infection, 9.78 × 10^6^ ± 1.12 × 10^6^ IFU/ml were recovered from syntaxin 10 siRNA-treated cells (Figure 2A). Infectious progeny are not included for NT siRNA-treated monolayers at 67 h post-infection because of the monolayer being destroyed by the end of the typical chlamydial developmental cycle; only ~21% of the monolayer remained intact (quantified by trypan blue exclusion assay, but also apparent in GAPDH levels, Figure 2B). The syntaxin 10 knockdown was 90.35% of control at 67 h post-infection (~113 h post-transfection), indicating that the slight increase (1.98-fold) in infectious progeny was not due to waning knockdown (Figure 2B). Notably, with an additional 23 h of culture, the organisms grown in syntaxin 10 siRNA-treated cells never displayed the same rate of growth or output levels as organisms grown in control cells. Further, syntaxin 10 knockdown monolayers, were largely intact at 67 h post-infection, in contrast to the control cells. These data support the notion that loss of syntaxin 10 results in a defect or delay in the chlamydial developmental cycle.

**Figure 3:**
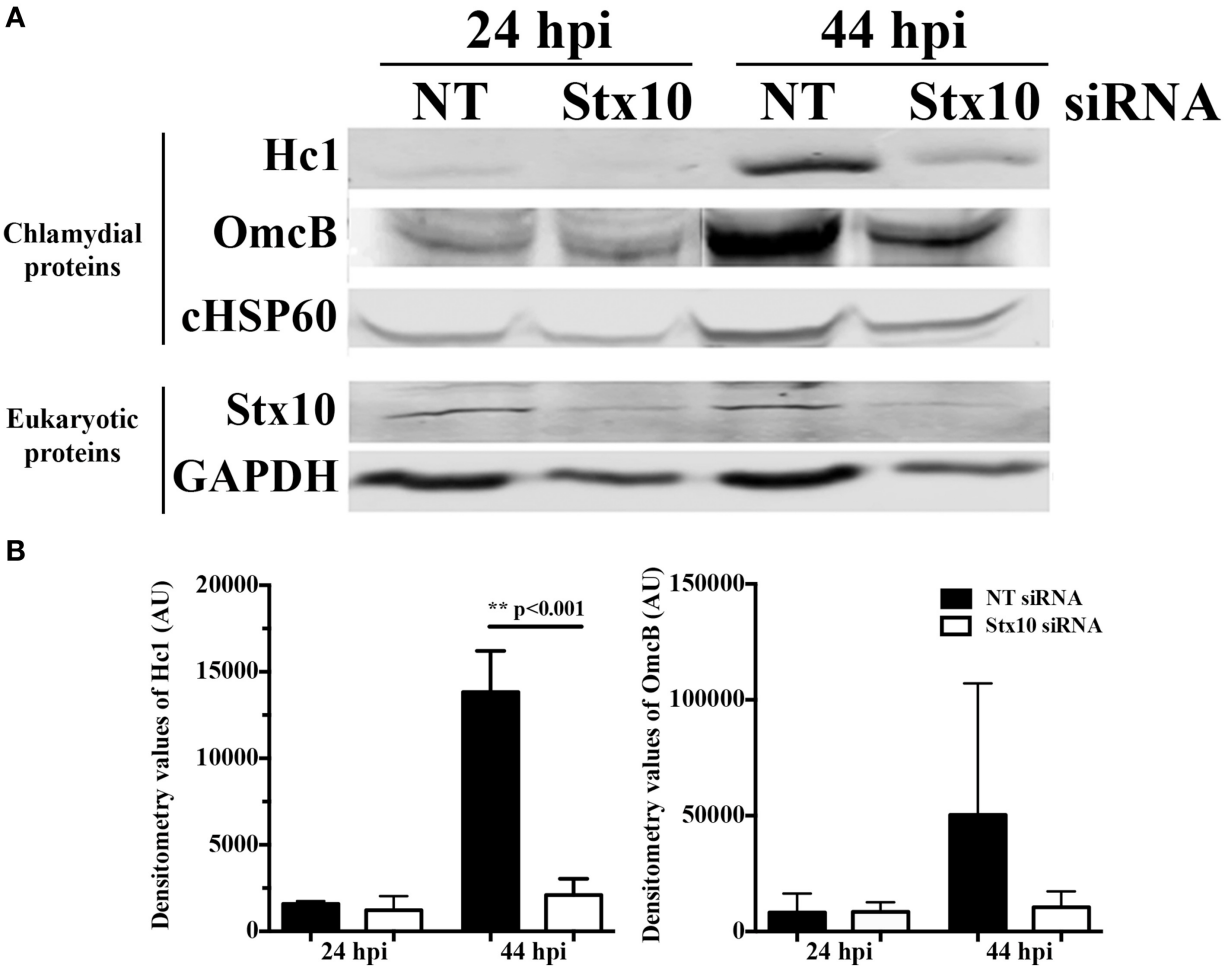
**Effect of siRNA knockdown of syntaxin 10 on expression of late chlamydial proteins: Hc1 and OmcB**. Syntaxin 10 or non-targeting (NT) siRNA-treated HeLa cells were infected with *C. trachomatis* serovar L2 for 24 or 44 h, prior to lysis in Laemmli sample buffer. **(A)** Equal amounts of protein were loaded onto SDS-PAGE gels and transferred prior to Western blot analysis using antibodies against chlamydial proteins Hc1, OmcB or chlamydial heat shock protein 60 (cHSP60) or eukaryotic proteins syntaxin 10 and GAPDH. The Western images shown are representative of two independent experiments. **(B)** Densitometry analysis was performed on the two independent experiments referred to in **(A)**. These values [expressed in arbitrary units (AU)] were obtained using the Image Studio imaging software associated with the LI-COR Odyssey Infrared Imaging System and then graphed as mean and standard error of the mean. Statistical analysis included an ordinary One-Way ANOVA with Tukey's multiple comparison test in GraphPad Prism 6 software.

**Figure 4:**
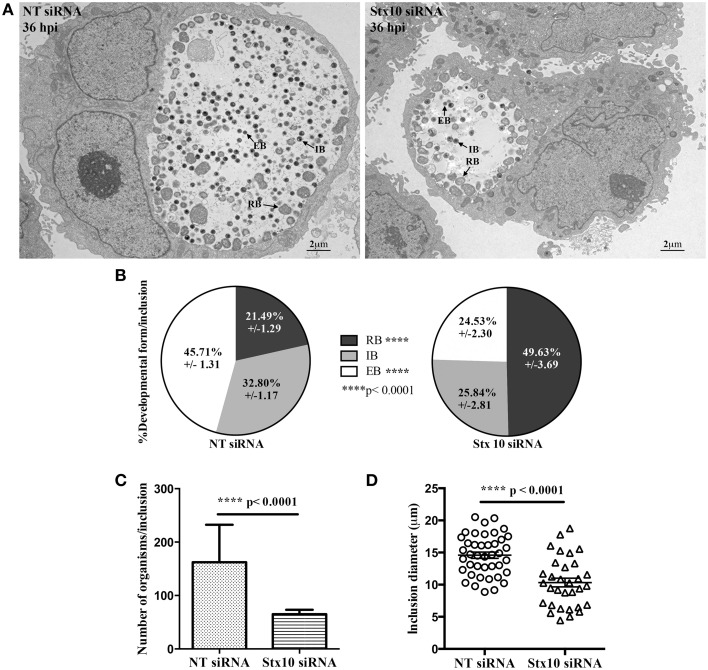
**Transmission electron micrograph analysis of the effect of syntaxin 10 siRNA knockdown on chlamydial development**. Syntaxin 10 (Stx10) or non-targeting (NT) siRNA-treated HeLa cells were infected with *C. trachomatis* serovar L2 for 36 h and then processed for transmission electron microscopy as described in Materials and Methods. For these studies, 40 images each from either Stx10 or NT-siRNA treated cells from 2 independent experiments were examined. Representative images are shown in **(A)** and Supplemental Figure 1. These images were used to quantify chlamydial developmental forms **(B)**, total numbers of organisms per inclusion **(C)**, or inclusion diameter **(D)**. All values were graphed to display the mean and standard error of the mean using GraphPad Prism 6 software. Statistical analysis included ordinary One-Way ANOVA with Tukey's multiple comparison test **(B)** and Student's *t*-test **(C,D)**.

To further examine the mechanism behind the decrease in chlamydial infectious progeny recovered from syntaxin 10 siRNA-treated cells, we examined the protein levels of two late developmental cycle proteins, Hc1 (*hctA* product) and OmcB by Western blot analysis (Figure 3). Consistent with the organisms being in mid-developmental cycle, at 24 h post-infection, there are no quantifiable differences in Hc1 or OmcB protein levels between organisms cultivated in NT or syntaxin 10 siRNA-treated cells. However, at 44 h post-infection, we observed an increase in protein levels of Hc1 and OmcB in NT siRNA-treated cells only, although only the difference in Hc1 levels was statistically significant (Figure 3B). We also tested transcript levels of the early gene *euo* by quantitative PCR, as a means to determine if we were seeing aberrant chlamydial development where *euo* transcript is elevated (see e.g., Ouellette et al., 2006). At 24 h post-infection, *euo* levels are approaching basal levels of transcription (Ouellette et al., 2014), and we found transcript levels increased by only 1.5 fold in organisms grown in syntaxin 10 siRNA-treated cells as compared to organisms grown in NT siRNA-treated cells (data not shown). While these data are statically significant, we do not consider these differences to be biologically significant and they indicate that the chlamydiae are not in a persistent growth state (see also Figure 4A and Supplemental Figure 3). We noticed a similar difference when examining *omcB* transcripts at 44-h post-infection (data not shown). Collectively, these data support that the developmental cycle of chlamydial organisms grown in syntaxin 10 knockdown cells is delayed or otherwise negatively impacted.

During the later stages of the chlamydial developmental cycle, the RB either continues to divide or begins to asynchronously redifferentiate into the infectious EB, forming the intermediate body (IB); (Ward, 1988). To confirm that loss of syntaxin 10 contributed to a delay in the chlamydial developmental cycle, we performed transmission electron microscopy (TEM) at 36 h post-infection in NT and syntaxin 10 siRNA-treated cells. Immediately apparent was the difference in both the size of inclusions and types of organisms found in NT siRNA and syntaxin 10 siRNA-treated cells (representative images, Figure 4A and Supplemental Figure 3). When we quantified the different bacterial developmental forms of *C. trachomatis* serovar L2 seen in the TEM images, organisms grown in syntaxin 10 siRNA-treated cells were more likely to be RBs than infectious EBs compared to organisms cultured in NT siRNA-treated cells. Similar percentages of IBs were obtained between experimental groups. Specifically, inclusions grown in NT siRNA-treated cells contained 45.71 ± 1.31% EBs, 32.80 ± 1/17 IBs, and 21.49 ± 1.29% RBs. Inclusions cultivated in syntaxin 10 siRNA-treated cells contained 24.53 ± 2.30 EBs, 25.84 ± 2.81 IBs, and 49.63 ± 3.69 RBs (Figure 4B). Notably, the overall number of organisms per inclusion was reduced when the bacteria were grown in syntaxin 10 siRNA-treated cells (65.10 ± 8.141 bacteria/inclusion) as compared to organisms grown in NT siRNA-treated cells (162.2 ± 10.98 bacteria/inclusion); (Figure 4C). Further, the average diameter of an inclusion grown in syntaxin 10 knockdown conditions was 10.33 μm (±0.6799 μm) compared to 14.59 μm (±0.4905 μm) in NT siRNA-treated cells (Figure 4D). Overall, these data indicate that in the absence of syntaxin 10, there is a negative impact on chlamydial inclusion size and chlamydial growth and development. Combined, these factors likely contribute to the decrease and delay in production of infectious progeny.

### Lipid trafficking to inclusions grown in syntaxin 10 knockdown cells

**Figure 5:**
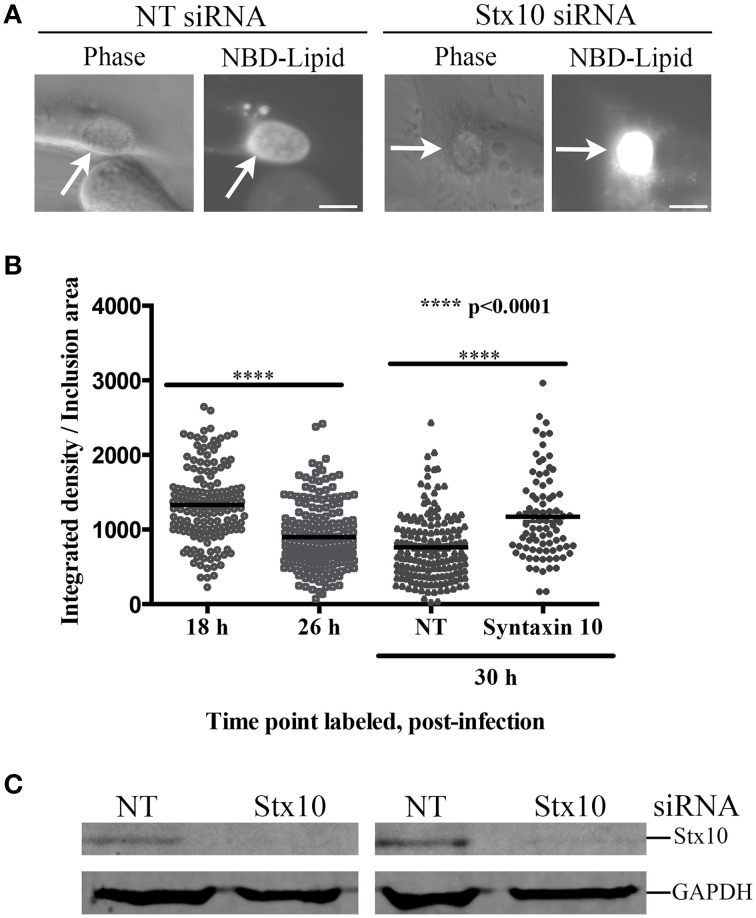
**Effect of syntaxin 10 siRNA knockdown on NBD-sphingomyelin trafficking to the chlamydial inclusion**. **(A)** Syntaxin 10 (Stx10) or non-targeting (NT) siRNA-treated HeLa cells were infected with *C. trachomatis* serovar L2 for 24 h, labeled with NBD-ceramide, and treated with back-exchange medium for an additional 3 h. Samples were imaged at 40X magnification with a 200 ms exposure time using an Axiovert 200 M Imager with the AxioCam HRm camera (Carl Zeiss Microscopy, LLC). These images are representative of 3 independent experiments. **(B)** Untreated HeLa cells were infected with *C. trachomatis* serovar L2 for 18 or 26 h, labeled with NBD-ceramide and back-exchanged for 1.5 h. In addition, Stx10 or NT siRNA-treated HeLa cells were infected with *C. trachomatis* serovar L2 for 30 h, labeled and back-exchanged as 18- and 26-h samples. Samples from two independent experiments were imaged using 60X magnification Olympus BX 60 fluorescent scope and images taken with a Nikon DS-Qi1Mc camera, with a 40 ms exposure for 18- 26- and NT siRNA treated samples, and a 20 ms exposure time for syntaxin 10-treated samples, as described in Materials and Methods. Individual data points, including the mean and standard error of the mean, were graphed using GraphPad Prism 6 software. Statistical analysis included an ordinary One-Way ANOVA with a Tukey's multiple comparison test. Representative images used in the quantitation are provided in Supplemental Figure 4. **(C)** A representative Western blot to demonstrate efficient knockdown of Stx10 compared to the loading control GAPDH.

We reasoned that defects to chlamydial maturation in the absence of syntaxin 10 might negatively correlate with chlamydial acquisition of host-derived nutrients. To examine this, we monitored sphingomyelin trafficking, a well-established marker of lipid trafficking in chlamydial infected cells, to inclusions by treating infected siRNA-treated monolayers with C_6_-NBD-ceramide and examined inclusions by live cell imaging. We observed that inclusions growing in syntaxin 10 siRNA-treated cells (Western blot analysis of knockdown efficiency shown in Figure 5C) retained substantially higher amounts of fluorescent lipid than in inclusions grown in NT siRNA-treated cells, when imaged at equivalent exposures (Figure 5A). To test if syntaxin 10 knockdown in HeLa cells caused overall retention of lipid within the Golgi and hence, by default, greater lipid retention within chlamydial inclusions, we examined uninfected cells by live cell imaging. After 4 h of back-exchange, there were no differences in remaining cell-associated fluorescence between non-targeting (control) and syntaxin 10 siRNA treated cells (data not shown). Additionally, TLC analysis of mock-infected HeLa cells treated with either syntaxin 10 or non-targeting siRNA demonstrated no difference in lipid retention (cell extracts)/trafficking (back-exchange medium); (data not shown); therefore, these results indicate that the increase in NBD-lipid in the chlamydial inclusion was not due to a general trafficking defect caused by syntaxin 10 knockdown. Further, we confirmed by TLC that the lipid species incorporated into purified chlamydial organisms was only NBD-sphingomyelin, indicating that the increase in fluorescence was not due to an alternative NBD-lipid product (Supplemental Figure 5).

We had difficulty in quantifying the amount of NBD-sphingomyelin retained in chlamydial organisms grown in syntaxin 10 siRNA-treated cells given the diversity of developmental forms. Therefore, we asked the question whether inclusions containing mostly RBs (e.g., 18 h post-infection) labeled with greater intensity than inclusions containing more EBs (e.g., 26–30 h post-infection). For these studies we infected HeLa monolayers for 18 or 26 h, labeled the cells for 20 min with C_6_-NBD-ceramide followed by a 1.5 h back-exchange period. 18-h inclusions demonstrated a mean of 1331 ± 39.08 (in arbitrary units) brightness per area of inclusion compared to 26-h inclusions, which demonstrated a mean of 899 ± 31.69 (in arbitrary units) brightness per area of inclusion (Figure 5B and Supplemental Figure 4). These data indicate that inclusions filled with mostly RBs are in essence “brighter” than more mature inclusions. Consistent with these data, 30-h inclusions grown in NT siRNA-treated cells displayed a mean of 759.6 ± 34.59 integrated density (arbitrary units) per inclusion area. In comparison, 30-h inclusions grown in syntaxin 10 siRNA-treated cells displayed a mean of 1172 ± 62.19 integrated density (arbitrary units) per inclusion area. Hence, less mature inclusions, or inclusions containing more RBs than EBs, are associated with greater fluorescence per area of the inclusion. Further, these data are consistent with the notion that loss of syntaxin 10 negatively impacts chlamydial growth and development, which correlates with altered nutrient acquisition.

## Discussion

In this study, we demonstrate that 3XFLAG-syntaxin 10-positive structures and/or 3XFLAG-syntaxin 10 alone localizes to the chlamydial inclusion (Figure 1). To investigate the role of syntaxin 10 at the inclusion, we depleted syntaxin 10 using siRNA knockdown. These studies revealed that loss of syntaxin 10 correlates with a significant delay in the progression of the chlamydial developmental cycle (Figure 2A). Further, loss of syntaxin 10 was associated with reduced expression of OmcB and Hc1 (Figure 3), late chlamydial proteins required for RB to EB differentiation (Hatch et al., 1986; Newhall, 1988; Mygind et al., 1998; Grieshaber et al., 2006). These results are consistent with TEM analysis demonstrating that the predominant developmental form at 36 h post-infection in syntaxin 10 siRNA-treated cells is the RB, compared to the EB found in control NT siRNA-treated cells (Figure 4). Further, 30-h inclusions cultivated in syntaxin 10 siRNA-treated cells retained similar amounts of lipids as more immature inclusions formed at 18 h post-infection in untreated HeLa cells (Figure 5). Given the heterogeneity of an siRNA knockdown, these results are all the more striking. Based on these data, we hypothesize that syntaxin 10-positive structures or syntaxin 10 localizes to the chlamydial inclusion for the purpose of contributing to and/or maintaining an environment conducive for optimal chlamydial growth and development by recruiting Golgi-derived vesicles or machinery to the chlamydial inclusion.

There are five general steps in the chlamydial developmental cycle: attachment and entry, EB-RB differentiation, RB cell division, RB-EB differentiation, and release of EBs to infect neighboring cells (Ward, 1988). Further, genomic profiling of expression of chlamydial genes has characterized when specific genes are expressed during the chlamydial developmental cycle, suggesting that the expression of certain genes correlates with optimal chlamydial development (Belland et al., 2003; Nicholson et al., 2003). In our studies, we found that two late proteins, associated with RB to EB differentiation, were poorly expressed in chlamydiae grown in syntaxin 10 siRNA-treated cells (Figure 3). In conjunction with the TEM data, loss of syntaxin 10 correlated with a defect or delay in chlamydial development, ultimately resulting in fewer infectious progeny. Given that syntaxin 10 plays a role in vesicle fusion, we originally hypothesized that loss of syntaxin 10 would result in mistrafficking or altered acquisition of a key metabolite, which would negatively impact chlamydial development. In this context, the loss of syntaxin 10 could lead to two non-mutually exclusive outcomes: erroneous trafficking of a molecular signal(s), which induces proper chlamydial growth and development, or loss of direct association between Golgi structures and the chlamydial inclusion. Our current studies are unable to distinguish whether syntaxin 10 has a direct effect (e.g., via interaction with an inclusion membrane protein) or syntaxin 10 is merely a marker for a subset of vesicles that provide *C. trachomatis* with a necessary nutrient. We are currently examining the syntaxin 10 and Golgi proteins that are binding partners of syntaxin 10 to characterize their localization in higher resolution in order to understand their exact roles in contributing to the optimal chlamydial growth environment. Ultimately, these studies will help us understand previously unidentified molecular triggers that impact chlamydial growth and development.

Given their reduced genome and incomplete metabolic pathways, RBs must scavenge nutrients and metabolites from the host in order to replicate. Even though the inclusion membrane segregates chlamydial organisms from the host cytosol, RBs interact extensively with the host cell through the inclusion membrane (Moore and Ouellette, 2014). For example, it is well established that chlamydiae utilize multiple, redundant pathways and mechanisms to selectively acquire host cell lipids (Hackstadt et al., 1996; Beatty, 2006; Heuer et al., 2009; Capmany and Damiani, 2010; Derré et al., 2011; Elwell et al., 2011; Cox et al., 2012; Kabeiseman et al., 2013; Boncompain et al., 2014). It is likely that the organisms are also simultaneously acquiring other metabolites, including the “molecular trigger” that prompts RB to EB differentiation, when acquiring host-derived lipids. Potentially impacting chlamydial growth and development is the smaller sizes of the chlamydial inclusions formed in syntaxin 10 siRNA-treated cells (Figure 4D). Based on the data demonstrating loss of syntaxin 10 resulted in decreased infectious progeny and smaller inclusions, we anticipated that loss of syntaxin 10 would negatively affect chlamydial acquisition of host lipids, including host-derived sphingomyelin. This hypothesis is consistent with previous data from the laboratory demonstrating that knockdown of VAMP4, a trans-Golgi SNARE that traffics to the inclusion, results in fewer infectious progeny and a disruption of lipid trafficking to the chlamydial inclusion (Kabeiseman et al., 2013). However, live cell imaging results contradicted this hypothesis (Figure 5). Using NBD-ceramide as a proxy to study Golgi-derived lipid trafficking to the chlamydial inclusion, we found that knockdown of syntaxin 10 resulted in “brighter” inclusions than what was observed in control siRNA-treated cells at the same time point. Interestingly, 30-h chlamydial inclusions grown in syntaxin 10 siRNA-treated cells retain similar amounts of lipid as 18-h inclusions grown in untreated HeLa cells (Figure 5B). Combined, these data suggest that sphingomyelin trafficking to the inclusion is not hindered by the loss of syntaxin 10, and, further, the presence of more RBs correlates with “greater” retention of fluorescent lipid. These results primarily support the conclusion that increased lipid retention is consistent with the abundance of immature developmental forms associated with growth in syntaxin 10 knockdown cells and not due to a trafficking defect of sphingomyelin caused by the absence of syntaxin 10.

There are two events that likely occur during ceramide-derived sphingomyelin labeling of chlamydiae: trafficking to the inclusion from the ER (and subsequently through the Golgi) and retention by the organism (Hackstadt et al., 1995, 1996; Scidmore et al., 1996). Prior work from Scidmore et al. (Scidmore et al., 1996) demonstrated that sphingomyelin trafficking to the inclusion was blocked in the presence of chloramphenicol when the antibiotic was added at 2–4 h post-infection, demonstrating a role for chlamydial protein synthesis in this process. The factors involved in retention of lipid are not clearly defined. Our Stx10 knockdown data indicate that Stx10 does not function in sphingomyelin trafficking to or retention by chlamydiae within the inclusion and suggests that sphingomyelin is trafficked through a specific subset of vesicles not characterized by this marker (syntaxin 10). Another previous study linked sphingomyelin acquisition to the progression of chlamydiae through the developmental cycle (Robertson et al., 2009). Our data suggest that there are additional metabolites that are necessary for chlamydiae to progress through the developmental cycle. This is consistent with other data demonstrating that the inclusion interacts with specific subsets of exocytic vesicles and will aid in our understanding of what nutrients and markers are delivered to the inclusion and by what route.

What is not ruled out by these studies is the possibility that the abundance of immature chlamydiae, due to the loss of syntaxin 10, would create a perpetual imbalance of chlamydial lipid acquisition to further stall the progress of chlamydial development. Previous studies have demonstrated that an over-accumulation of a nutrient at the chlamydial inclusion resulted in negative effects on chlamydial development (Ouellette and Carabeo, 2010). Hence, an imbalance in the amount of lipid in inclusions grown in syntaxin 10 siRNA-treated cells may contribute to defects in the differentiation of RBs to EBs in the later stages of chlamydial development, ultimately contributing to lower yields of EBs (Figure 2A). RBs have a fragile cell wall, where an overabundance of a specific lipid may alter their membrane fluidity. Accumulation of sphingomyelin in chlamydial cell membranes will likely stiffen the bacterial membranes since sphingomyelin contains hydrophobic chains, which tend to be much more saturated than other phospholipids (Barenholz and Thompson, 1980; Van Blitterswijk et al., 1981; Chiu et al., 2003). Excessive sphingomyelin may prevent the chlamydial cell membrane from being repackaged properly, which would potentially lead to defects in differentiation at late stages of chlamydial development, inefficient division, or a reduction in growth rate. Unfortunately, our data do not distinguish between “cause” and “effect.” Specifically, we cannot definitively conclude that the lack of progression through the developmental cycle of *Chlamydia* grown in syntaxin 10 siRNA-treated cells is due to excess lipid accumulating within the inclusion as opposed to the distinct conclusion that immature developmental forms naturally retain “more” lipid given their greater membrane surface area compared to mature EBs.

## Concluding remarks

For the first time, we demonstrate that trans-Golgi SNARE protein syntaxin 10-positive structures and/or syntaxin 10 localizes to the chlamydial inclusion. Further, syntaxin 10 is utilized at the chlamydial inclusion to support optimal growth and development, as loss of syntaxin 10 results in significant delays in production of chlamydial progeny.

### Conflict of interest statement

The authors declare that the research was conducted in the absence of any commercial or financial relationships that could be construed as a potential conflict of interest.
