# Supplementary material for: The trans-Golgi SNARE syntaxin 10 is required for optimal development of Chlamydia trachomatis
Source: Front Cell Infect Microbiol. 2015 Sep 25;5:68. doi: 10.3389/fcimb.2015.00068 (PMC4585193; doi:10.3389/fcimb.2015.00068)
Supplement: Supplementary file 1 [file DataSheet1.DOCX]

**Supplemental Figure 1. Localization of syntaxin 10 to the chlamydial inclusion.** HeLa cells were transfected with 3XFLAG-syntaxin 10 (3XF-stx10) and infected for 8 hrs (A), 13 hrs (B) or 36 hrs (C) and processed for imaging as in Figure 1B. Samples that were treated with brefeldin A (BFA), were treated with 1 μg/ml for 2 hours prior to fixation. Samples that were treated with 200 μg/ml chloramphenicol for 22 hours, then BFA was added to the wells for an additional 2 hours prior to fixation. In A, the inclusions are very small and did not react with the anti-IncG antibody. Hence, inclusions are indicated by white dotted circles surrounding the area with the inclusions at the center of the circle (organisms stained with DAPI in blue). In B and C, inclusions are indicated with an asterisk. Scale bars are equivalent to 10 μm. As noted in the text, it is difficult to determine whether 3XFLAG-syntaxin 10 localizes to the inclusion at early time points (8 hours) post-infection.

**Supplemental Figure 2. Golgi structure in syntaxin 10 knockdown cells. A.** HeLa cells were treated with syntaxin 10 siRNA or non-targeting control siRNA and infected with *Chlamydia* for 16-18 hours. Cells were processed for indirect immunofluorescence to detect *Chlamydia* (blue) and giantin (Golgi, green). The results shown are representative images of at least 3 independent experiments. Scale bars = 10μm. **B.** HeLa cell lysates were collected in lysis buffer after siRNA transfection and infection. Western blot analysis was completed to detect syntaxin 10 and GAPDH in order to verify knockdown efficiency. In syntaxin 10 knockdown cells the Golgi structure is disrupted and does not localize to inclusions compared to control cells.

**Supplemental Figure 3. Collection of transmission electron micrograph images demonstrating the effect of syntaxin 10 siRNA knockdown on chlamydial development. A.** A depiction of the developmental forms analyzed: the division-competent reticulate body, intermediate body (representative of the intermediate form during reticulate body redifferentiation into the elementary body), and the infectious elementary body. **B.** Representative images of 36 hours post-infection inclusions found in control or HeLa cells treated with non-targeting siRNA versus HeLa cells treated with syntaxin 10 siRNA. In general, at 36 hours, inclusions found in non-targeting siRNA-treated cells were dominated by elementary bodies, while a few dividing reticulate bodies were seen at the periphery of the inclusion, juxtaposed to the chlamydial inclusion membrane. In contrast, inclusions that formed in syntaxin 10 siRNA-treated cells demonstrated a diversity of phenotypes, but were typically smaller in size and dominated by reticulate bodies. In some instances, the inclusion membrane was not apparent (bottom row, middle panel). It is unclear if the inclusion membrane is more fragile in syntaxin 10 knockdown cells or if this is a result of sample processing. Because of this uncertainty, these inclusions were eliminated from further analysis.

**Supplemental Figure 4. Sample fields of view used to quantitate retention of NBD-sphingomyelin in the chlamydial inclusion.** Time of infection of untreated cells or siRNA transfection before infection with *C. trachomatis* serovar L2 for 30 hours are indicated. Additionally, the exposure time, expressed in milliseconds (ms) is indicated. These images contributed to the quantitation seen in Figure 5B and were prepared as described in the *Materials and Methods*.

**Supplementary Figure 5. Incorporation of lipids in chlamydial organisms grown in syntaxin 10 knockdown cells. A.** HeLa cells were treated with syntaxin 10 siRNA or non-targeting control siRNA and then infected with *C. trachomatis* serovar L2 for 40-44 hours. Cells were labeled with C_6_-NBD-ceramide, and back-exchanged overnight. EBs were purified after 40-44 hours of infection and lipids were extracted as previously described (Moore et al., 2008). The results shown are representative images of one out of three experiments. Sphingomyelin (SM); Lactosylceramide (LC); Glucosylceramide (GlCer); Ceramide (Cer). LC serves as a loading control and was added to the samples prior to lipid extraction. **B.** Western blot analysis was performed on mirror samples lysed in sample buffer to determine organism load and knockdown efficiency, respectively.
