# Supplementary figures and images for: The trans-Golgi SNARE syntaxin 10 is required for optimal development of Chlamydia trachomatis
Source: Front Cell Infect Microbiol. 2015 Sep 25;5:68. doi: 10.3389/fcimb.2015.00068 (PMC4585193; doi:10.3389/fcimb.2015.00068)

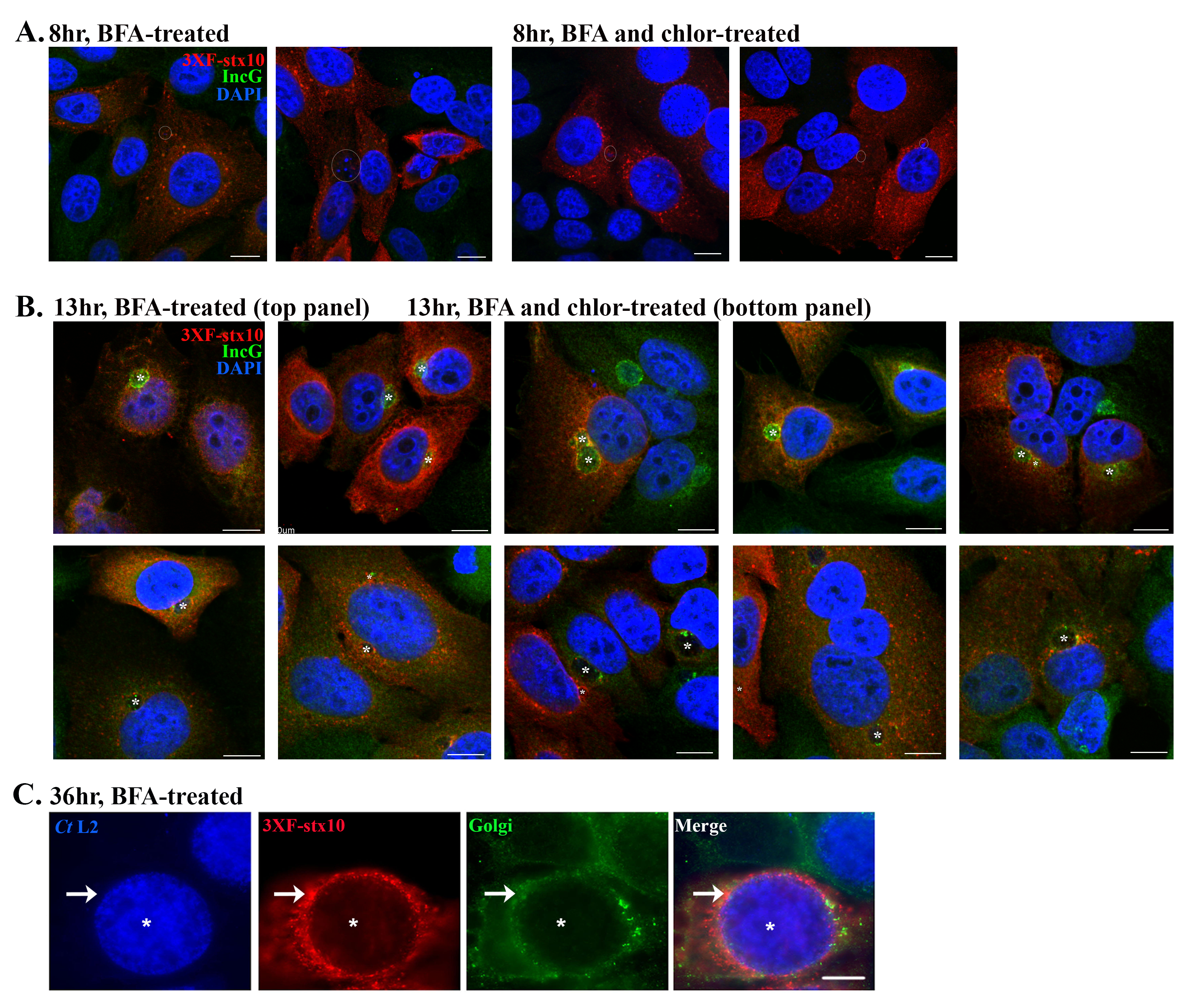

Supplement: Supplementary file 2 [file Image1.TIF]

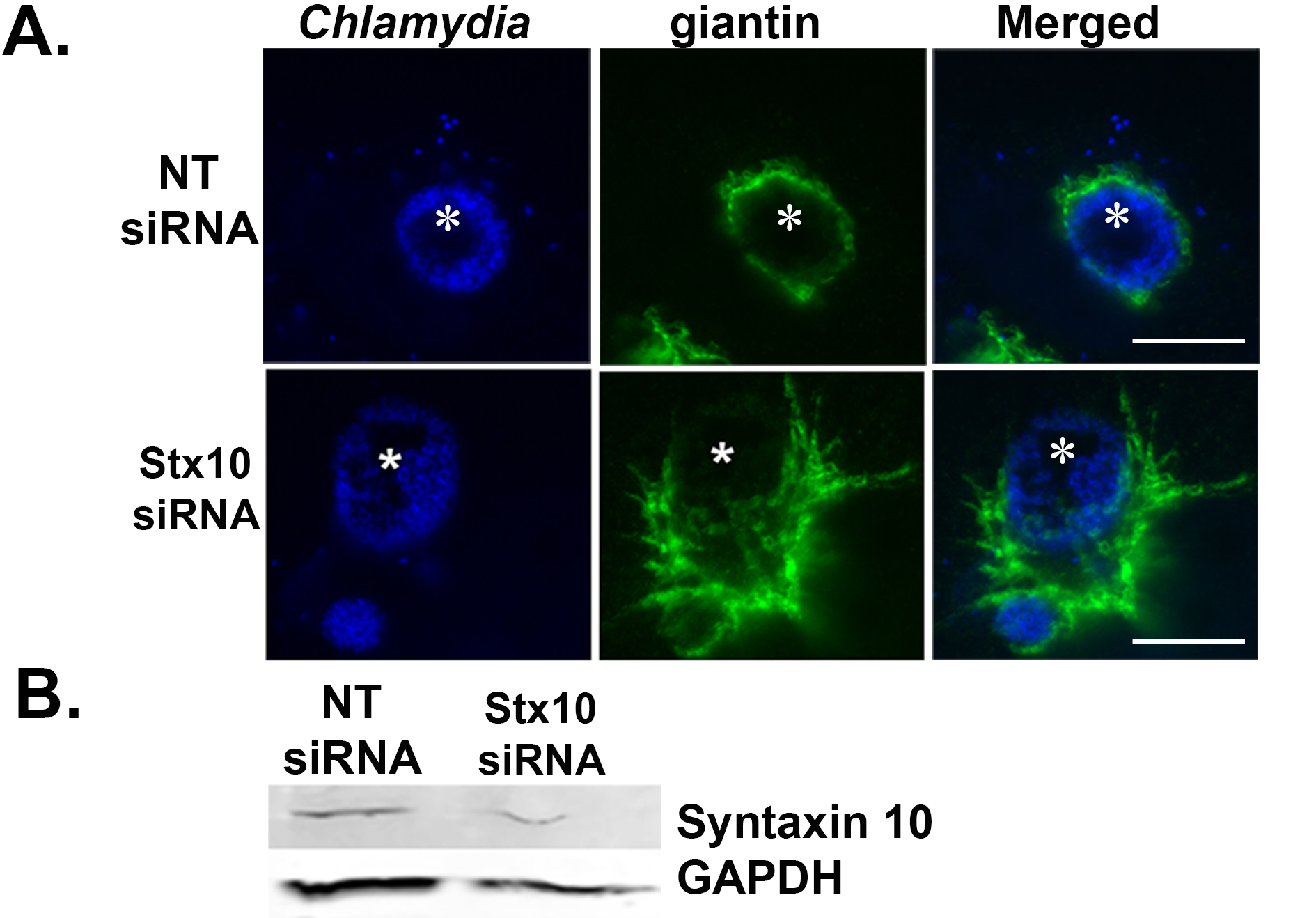

Supplement: Supplementary file 3 [file Image2.TIF]

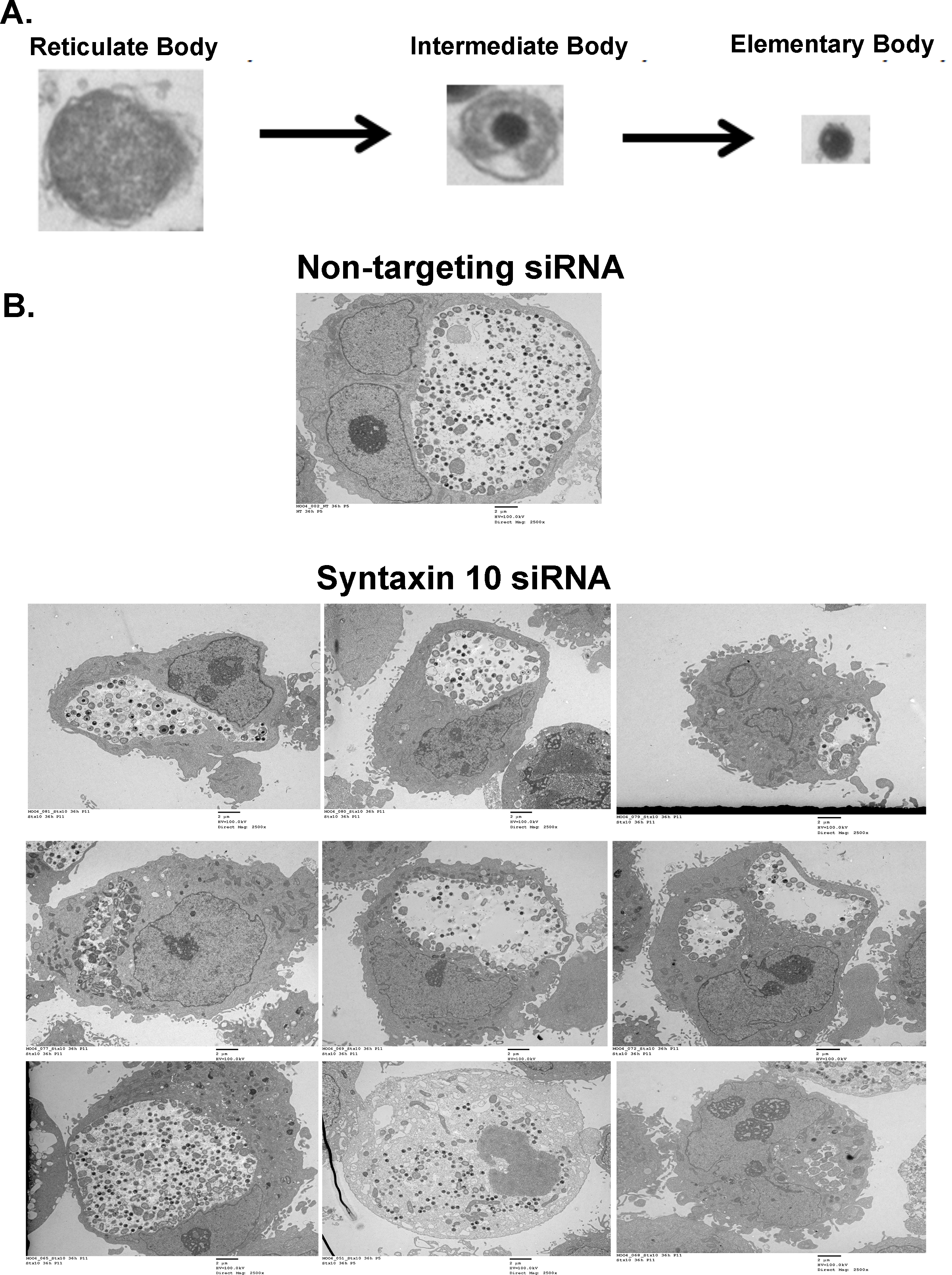

Supplement: Supplementary file 4 [file Image3.TIFF]

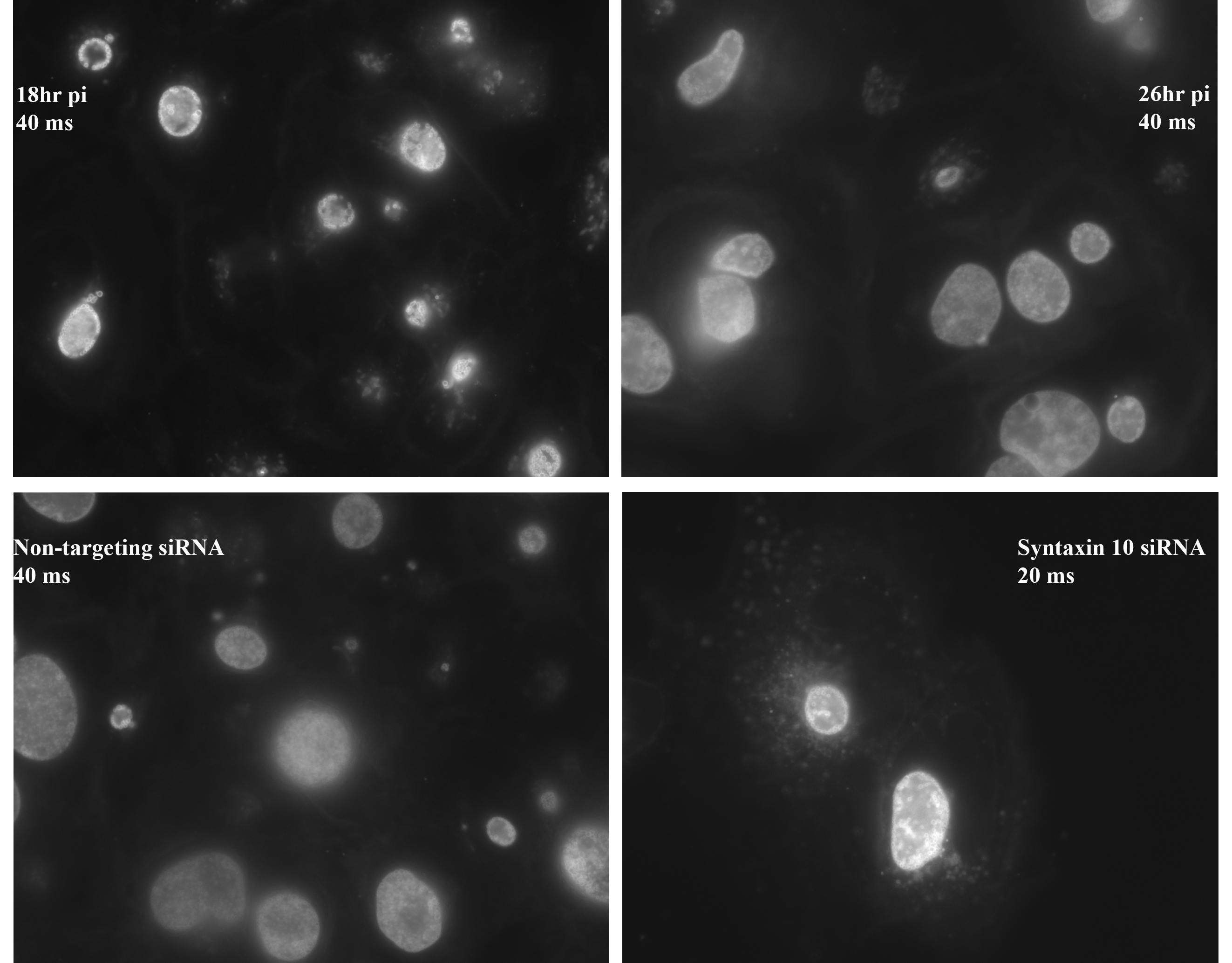

Supplement: Supplementary file 5 [file Image4.TIF]

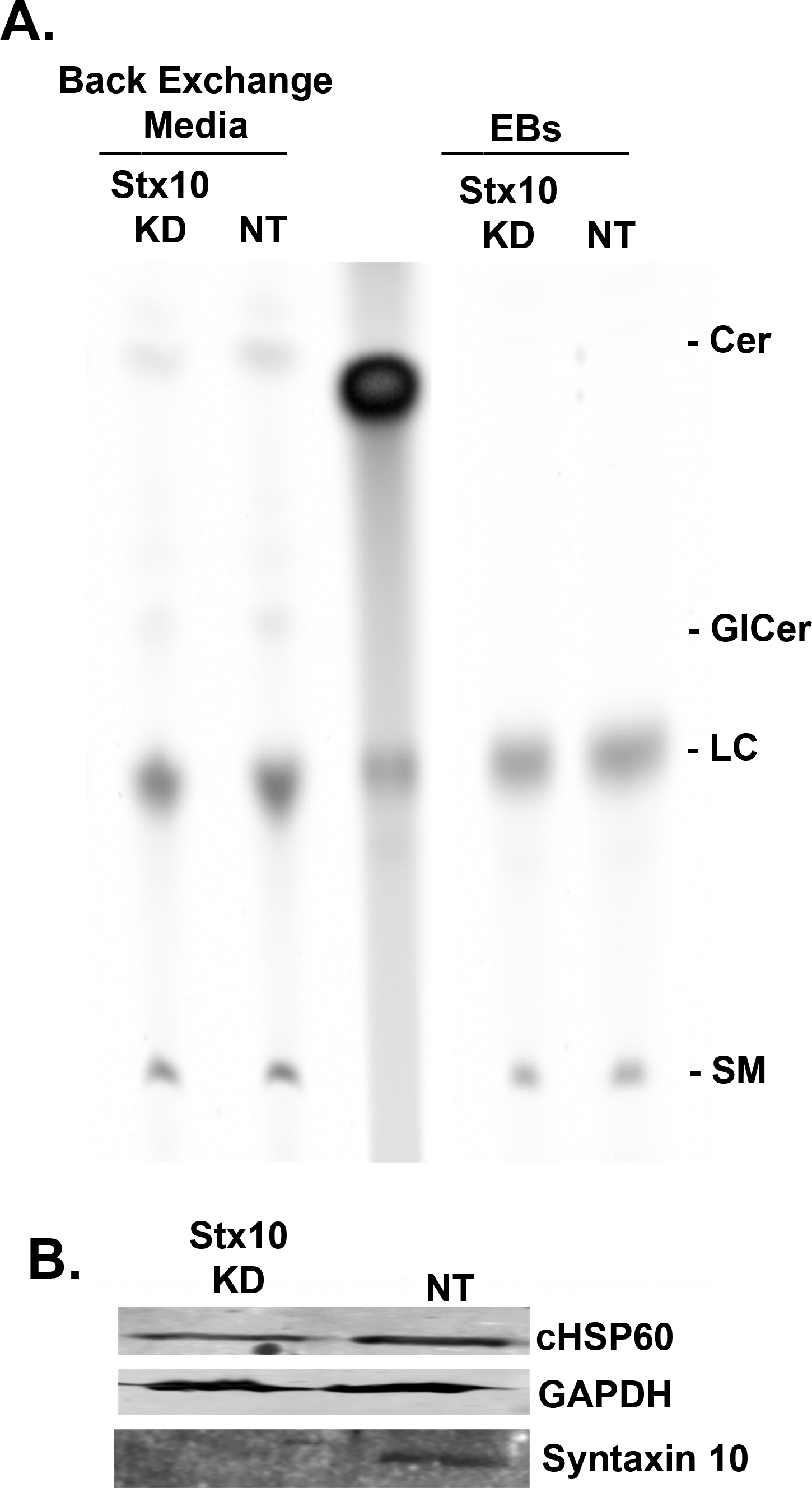

Supplement: Supplementary file 6 [file Image5.TIF]
